# Dual-Phase Mixed Protonic-Electronic Conducting Hydrogen Separation Membranes: A Review

**DOI:** 10.3390/membranes12070647

**Published:** 2022-06-24

**Authors:** Hongda Cheng

**Affiliations:** Zibo Vocational Institute, Zibo 255300, China; chd_98766@163.com

**Keywords:** dual-phase, membrane, mixed protonic-electronic conducting, hydrogen separation

## Abstract

Owing to the excellent properties of high selectivity, high thermal stability, and low cost, in the past twenty years, mixed protonic-electronic conducting hydrogen separation membranes have received extensive attention. In particular, dual-phase mixed protonic-electronic conducting membranes with high ambipolar conductivity are more attractive because of the high hydrogen permeability. This paper aimed to present a review of research activities on the dual-phase membranes, in which the components, the characteristics, and the performances of different dual-phase membranes are introduced. The key issues that affect the membrane performance such as the elimination of the inter-phase reaction, the combination mode of the phases, the phase ratio, and the membrane configuration were discussed. The current problems and future trends were simply recommended.

## 1. Introduction

Over the past twenty years, with the development of the green economy, hydrogen has attracted considerable attention as one of the most important renewable and clean energy sources [1,2,3]. Each year the hydrogen consumption exceeds sixty million tons in industry [4]. To meet the rapidly increasing demand for hydrogen, many technologies have been developed to produce hydrogen, such as water electrolysis, methane steam reforming, and ammonia decomposition [5]. However, no matter what technology is used, hydrogen separation is one indispensable step because of the presence of by-products. At present, membrane technology, cryogenic distillation and swing adsorption (PSA) are the main hydrogen separation methods; among these, membrane technology is considered to be more promising owing to the advantages of ease of operation, cost efficiency, and process intensification opportunity [6,7,8]. Reactors based on hydrogen separation membrane can simultaneously carry out reaction and hydrogen purification in one unit, saving equipment space and capital costs [9,10].

Based on different permeation mechanism, membrane for hydrogen separation can be categorized as mixed protonic–electronic conducting (MPEC) membrane, dense metal membrane, and porous inorganic membrane. Each membrane also has its own advantages and disadvantages. Porous inorganic membranes such as zeolite membranes relay on molecular sieving mechanism, which possess high hydrogen permeability and good chemically stability. However, the low selectivity of hydrogen permeation restricts their application, especially on occasions when high purity hydrogen is required [11,12]. Palladium (Pd) or palladium alloy membranes possess excellent hydrogen permeation performance, but their large-scale commercial applications still are limited by the low thermal stability, poisoning by gas impurities, and the high cost [13,14,15,16]. As a notable membrane technique, MPEC membranes with high thermal stability and suitable price exhibit great development prospect in fields of hydrogen separation membranes, hydrogen sensors, and fuel cells [17]. Perovskite oxides Ba(Sr)CeO_3_ and lanthanide tungstate oxides LnWO (Ln = La, Nd, In, Sm) are the main materials prepared for MPEC membrane, and the hydrogen transport is achieved by the conduction of protons and electrons; moreover, the theoretical selectivity of hydrogen permeation can reach 100% [18,19,20,21]. Taking into account these advantages, MPEC membrane is of great promise as an applicative and economical hydrogen separation membrane.

Wanger and Shores first discovered that some metal oxides exhibited proton transport property when exposed to hydrogen at high temperatures, but the protonic conductivity was so poor that they did not attract much attention. After 1981, when Iwahara reported that some perovskite oxides exhibited obvious proton transport characteristics at high temperatures (≥600 °C), mixed protonic–electronic conducting membranes began to be intensively studied [22]. The hydrogen permeation of MPEC membranes begins with the combination of the hydrogen atom and the membrane intrinsic defects [23]; therefore, the concentration of intrinsic defects, especially the concentration of oxygen vacancy, is critical to the hydrogen permeability of MPEC membranes, more oxygen vacancy means higher protonic conductivity. To increase the oxygen vacancy of the membrane, and aliovalent doping is an effective approach. For ABO_3_ perovskite oxides, partially substituting B cations with aliovalent cations can efficiently increase the amount of oxygen vacancy. The doped perovskite oxides show obvious higher protonic conductivity compared with those of un-doped in hydrogen or water vapor containing atmosphere. Until now, a lot of trivalent cations including Y, Pr, Tb, Yb, and Sm have been used to substitute the cation of Ce or Zr in BaCe(Zr)O_3_ membranes [24,25,26]. The chemical stability, hydrogen permeation flux, and electrical conductivity of the doped membranes also have been thoroughly investigated. Similar to perovskite oxides, the doping strategy for lanthanide tungstate oxides are mainly focus on the partial substitution of W with Ir, Re, Mo, or Nb [27,28,29].

High ambipolar conductivity is necessary for MPEC membranes to gain high hydrogen permeation fluxes. Compared with the relatively high protonic conductivity, the lack of sufficient electronic conductivity restricts the practical application of MPEC membranes. The electronic conductivity of protonic conductor can be enhanced by aliovalent doping. It is found that the properties of the dopant ions such as the ionization potential is related to the membrane electronic conductivity [30]. For example, the electronic conductivity of SrCeO_3_-based membranes in reducing atmosphere increases with the increase of the dopant ion ionization potential [31]. However, the improvement of electronic conductivity by aliovalent doping is finite; moreover, this strategy could be detrimental to the protonic transport of the membrane. For example, while the p-type conductivity of Y-doped SrCeO_3_ is proportional to Y concentration, the protonic transport ability of the material declines following the increase of Y [32].

In recent years, dual-phase MPEC hydrogen separation membranes with high electronic and protonic conductivity have attracted great attention. The dual-phase MPEC membrane is fabricated by combing protonic conductor with an electronic conducting phase; in addition, according to the composition difference of the electronic conducting phase, dual-phase MPEC membranes are further divided into ceramic–metal (cermet) membranes and ceramic–ceramic (cercer) membranes, in which metal and ceramic with high electronic conductivity are used respectively.

This review provides an overview on the current advances in dual-phase MPEC membranes. Electronic conductivity, hydrogen permeability, and membrane stability were summarized. The combination mode of the two phases and the membrane configuration were discussed. The current problems and future trends of dual-phase membrane were also discussed at the end.

## 2. Transport Mechanisms

**Figure 1:**
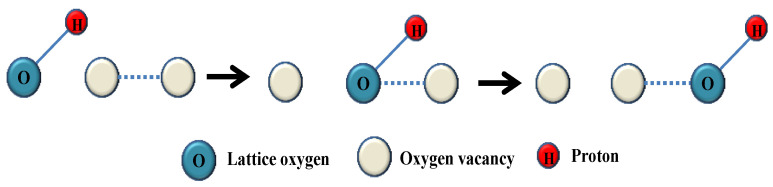
Schematic diagram of the bulk diffusion of protons through the membrane [17].

The hydrogen permeation through MPEC membrane includes three processes: the first is the gas–solid interface exchange at the membrane surface in the feed side, the second is the bulk diffusion of protons through the membrane, and the third is the gas–solid interface exchange at the membrane surface in the permeate side. The presence of a large number of oxygen vacancies in the membrane material is crucially important to the hydrogen permeability of MPEC membranes. The permeation of hydrogen begins with the combination of dry or wet hydrogen and the oxygen vacancies or lattice oxygen on the membrane surface, as shown in Equations (1) and (2). The formed hydroxide ion occupies the oxygen vacancy site and the proton attaches itself to the lattice oxygen. In both cases, the protons are combined with oxygen ions forming OH^−^. There are different theories about the proton diffusion through the membrane; according to the vehicle mechanism, the bulk diffusion of proton occurs through the hydroxide ions movement via oxygen vacancies, as shown in Figure 1.

(1) $$H_{2}{O(g)+V}_{O}^{••}{+O}_{O}^{\times }\;↔{\;2\mathrm{OH}}_{O}^{•}$$

(2) $$O_{O}^{\times }+\frac{1}{2}H_{2}\;↔{\;\mathrm{OH}}_{O}^{•}+e^{\prime }$$

where $V_{O}^{••}$ is the oxygen vacancy, $O_{O}^{\times }$ is the lattice oxygen, and ${\mathrm{OH}}_{O}^{•}$ is the hydroxide ion.

**Figure 2:**
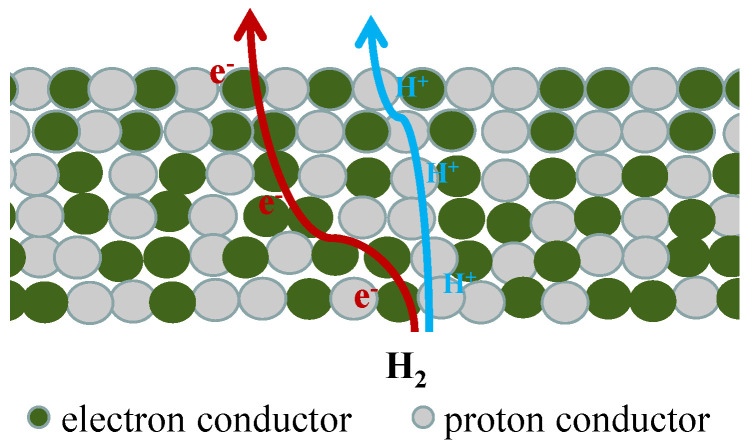
Schematic diagram of the transport of protons and electrons in dual-phase membranes.

For single-phase MPEC membranes, the relatively low electronic conductivity limits their hydrogen permeability. In dual-phase membranes, proton conductor is combined with electronic conductors with high electronic conductivity, and separate transport channels for protons and electrons are formed inside the membrane (Figure 2). Therefore, compared with single-phase membranes, dual-phase membranes are expected to have higher ambipolar conductivity and higher hydrogen permeability.

## 3. Cermet Dual-Phase Membrane 

Cermet membrane is composed of a ceramic phase and a metal phase. The ceramic phase is benefit to improve the mechanical stability and high protonic conductivity of the membrane while the metal phase is used to enhance the electronic conductivity and surface-exchange kinetic. Palladium (Pd) and nickel (Ni) are the common used metals in cermet membranes.

### 3.1. Pd-Based Cermet Membranes 

**Figure 3:**
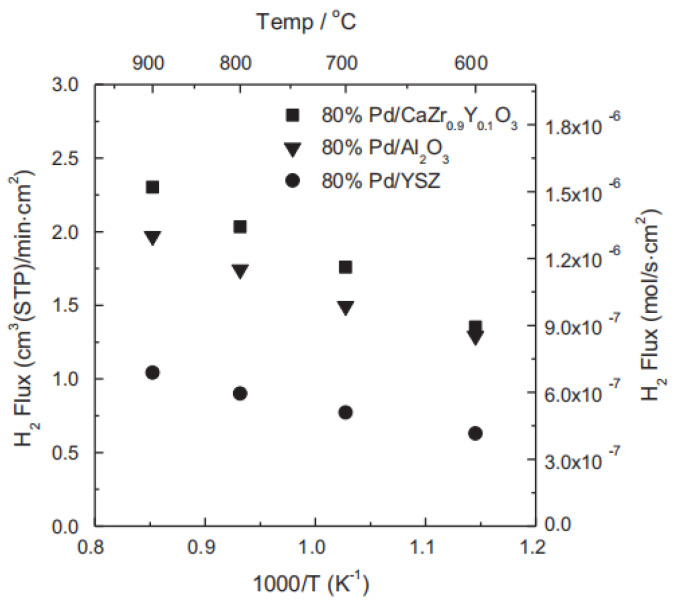
Three Pd-based cermet dual-phase membranes with different hydrogen permeation fluxes [34].

Dense Pd membrane is a kind of membrane with excellent hydrogen permeability, and it can be applied to prepare high purity and high flux hydrogen. However, the low mechanical stability, low chemical stability, and the high cost restrict its industry application. In 2006, Argonne National Laboratory developed a cermet dual-phase membrane using Pd as the hydrogen transport metal and yttria-stabilized ZrO_2_ (YSZ) as the mechanically stable matrix [33]. The membrane showed a high hydrogen permeability, of which the H_2_ permeation rate reached 20 mL·min^−1^·cm^−2^ at 900 °C. Moreover, the membrane stability was investigated under H_2_S atmosphere. Hydrogen flux showed no degradation for 270 h in atmosphere containing 400 ppm H_2_S. The results indicated the combination of hydrogen separation metal and ceramic matrix can ensure the high hydrogen permeability and good mechanical stability of the membrane. Inspired by the excellent performance of Pd-YSZ, Jeon et al. fabricated a cermet dual-phase membrane by embedding Pd in protonic conducting ceramic matrix CaZr_0.9_Y_0.1_O_3-δ_ (CZY) [34]. In this membrane, CZY is not only the matrix of Pd but also an active contributor for the hydrogen separation, facilitating the transport of protons through the membrane. Therefore, the permeation flux of this membrane consists of two parts: one is from the metal phase via atomic diffusion, another is from the ceramic phase via ambipolar diffusion (Equation (3)).

(3) $$J_{H_{2}}=-\left(\frac{\mathrm{xRT}}{{4\mathrm{LF}}^{2}}\int_{p_{H_{2}}^{s}}^{p_{H_{2}}^{f}}\frac{\sigma_{{\mathrm{OH}}_{O}^{•}\;}\sigma_{e^{\prime }}}{\sigma_{{\mathrm{OH}}_{O}^{•}}{+\sigma }_{e^{\prime }}}{\mathrm{dlnp}}_{H_{2}}+(1-x)\frac{\Phi }{L}\nabla p_{H_{2}}^{1/2}\right)$$

where σ_i_ is the partial conductivity of ${\mathrm{OH}}_{O}^{•}$ and e′, x is the volume fraction of CZY, L, F, and Φ are membrane thickness, Faraday constant, and hydrogen permeability of Pd respectively. The experiment results showed that the hydrogen flux of Pd-CZY had remarkable enhancement compared with that of Pd-YSZ and Pd-Al_2_O_3_ (Figure 3), and the flux ascribing to the ambipolar diffusion of CZY accounted for about 30% of the total flux. The combination of Pd and proton conductor is an efficient method to prepare cermet hydrogen permeation membranes. The study exhibited BaCeO_3_-based perovskite oxides possessed the highest protonic conductivity among all types of protonic conductors; based on this, Tsai et al. reported a cermet membrane composed of Pd and perovskite oxide-BaCe_0.4_Zr_0.4_Gd_0.1_Dy_0.1_O_3-x_ (BCZGD) [35], and the membrane had a higher hydrogen permeability than the Pd-CZY system. At 700 °C, the flux of the membrane with 400 µm thickness reached 1.25 mL·min^−1^·cm^−2^. Several representative cermet dual-phase membranes and their hydrogen permeation performances are listed in Table 1. 

Pd-based cermet membranes are one kind of advanced dual-phase membranes which combine high permeability, high selectivity, and good chemical stability successfully. Because the hydrogen permeation through these membranes is mainly via the hydrogen atom transport of Pd, the presence of Pd makes Pd-based cermet membranes exhibit much higher hydrogen permeability. In addition to affording additional ambipolar hydrogen permeation, the protonic conducting ceramic phase is mainly used as the matrix for the metallic phase, and to facilitate the chemical and thermodynamic stability of the membrane.

### 3.2. Ni-Based Cermet Membranes

Attributing to the unique properties, Ni is considered to be one of the most suitable metals used for conducting electrons in cermet membranes. As a well-known anode catalyst for fuel cells, Ni has high surface exchange kinetics, is cheaper, and has superior chemical and thermal stability compared with Pt. In addition, Ni has similar thermal expansion with BaCe_0.9_Y_0.1_O_3-δ_, which can enable Ni to have good compatibility with ceramic phases. Moreover, high sintering temperature is necessary to prepare a dense membrane, and the melting point of Ni is up to 1453 °C and can ensure the stability of the membrane during the sintering process. Meng et al. prepared a cermet dual-phase membrane via ball-milling Ni and BaCe_0.95_Tb_0.05_O_3-__α_ (BCTb) mixtures in a weight ratio of 1:1. The total conductivity of the membrane reached 400 S·cm^−1^ at room temperature, which was much higher than the value of single-phase BCTb membrane. The H_2_ permeation flux at 850 °C reached 0.53 mL·min^−1^·cm^−2^ [46]. 

**Figure 4:**
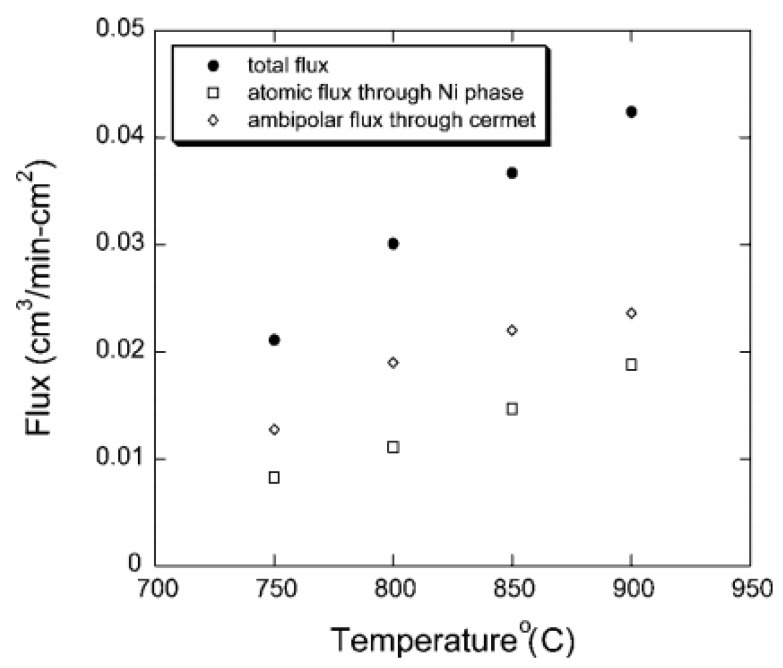
The contribution of two diffusion mechanisms to the total hydrogen permeation fluxes in Ni-SCYb cermet dual-phase membranes [52].

The content of the metal phase in the composites is closely associated with the hydrogen permeability. To gain high hydrogen permeability, the amount of the metal phase needed in the composites should be firstly ascertained. Kim et al. studied the effect of membrane composition on the membrane electrical conductivity and hydrogen permeability [48]. The results showed that only when the Ni content reached 40 vol%, the metal phase could form a three-dimensional network and cause sufficient Ni connectivity. The membrane containing 40 vol% Ni had higher electrical conductivity and H_2_ permeability compared with the membranes with 30 and 35 vol% Ni. As shown in Table 1, Ni content in most of the current Ni-based dual-phase membranes was set to 40 vol%. Song et al. prepared cermet composite Ni-SrCe_0.8_Yb_0.2_O_3-x_ (Ni-SCYb) containing 40 vol% Ni via ball-milling method. Both the membrane electronic conductivity and surface exchange kinetics were improved distinctly. The hydrogen flux was ten times that of single SCYb membrane. Hydrogen permeation due to the atomic diffusion through Ni phase was also detected (Figure 4). However, compared with Pd-based composite membranes, the atomic hydrogen diffusion of Ni is much weaker, and the ambipolar diffusion of SCYb was dominant over the whole temperature range [52]. 

Similar to Pd-based cermet membranes, the hydrogen permeability of Ni-based dual-phase membranes increases with the temperature rising. Since the electronic conductivity of the Ni phase decreases at elevated temperatures, enhancement of membrane permeability at high temperatures should be attributed to the improvement of the protonic conductivity of the ceramic phase. Therefore, for Ni-based dual-phase membranes, the hydrogen permeability mainly depends on the protonic conductivity of the ceramic phases. For the Ni phase, although the electronic conductivity decreases at high temperatures, the hydrogen permeability increases because of the improvement of the atomic diffusion.

**Figure 5:**
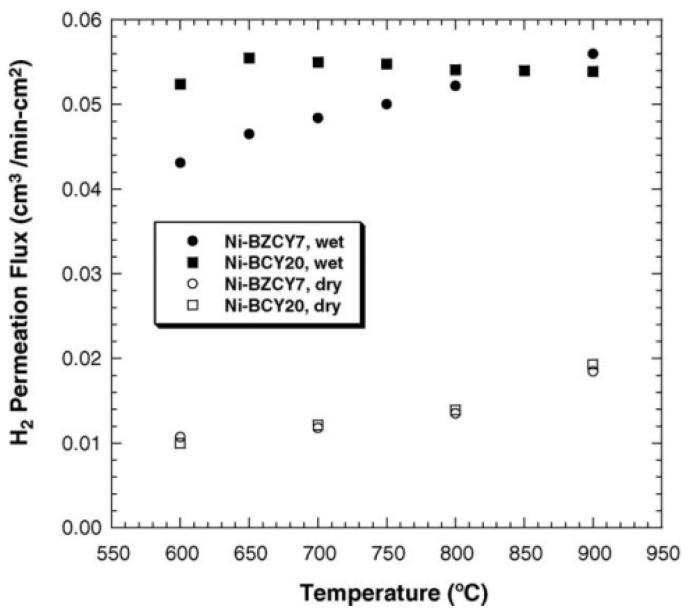
H_2_ fluxes through Ni-BZCYb membrane feeding with dry and wet 4% H_2_ [41].

**Figure 6:**
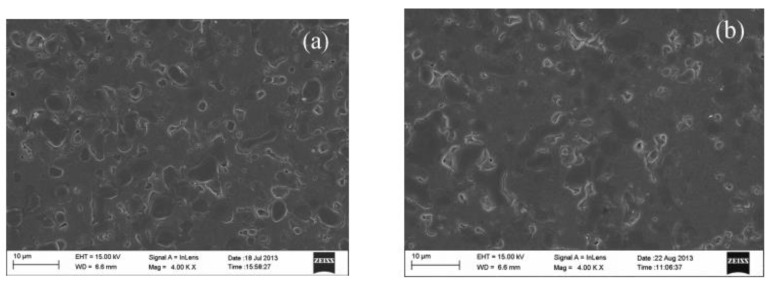
SEM images of Ni-BZCYYb sintered at 900 °C for 200 h in 20% CO_2_ containing atmosphere, (**a**) the original membrane, and (**b**) the sintered membrane [44].

The stability is important for hydrogen separation membranes, especially for dual-phase membranes under vapor or CO_2_-containing atmosphere. Humidity is favorable for the ceramic phase; both the catalytic activity and protonic conductivity of the ceramic phase will be enhanced owning to the hydration of the membrane. However, humidity is unfavorable for the metal phase; the conductivity of the metal phase will decrease in wet atmosphere due to the oxidizing of the surface. Zuo et al. studied the hydrogen permeability of Ni-Ba(Zr_0.8-x_Ce_x_Yb_0.2_)O_3-α_ (Ni-BZCYb) membrane under wet conditions. As shown in Figure 5, Ni-BZCYb had higher hydrogen fluxes under wet conditions [41]. The stability of composite membrane Ni-BaZr_0.1_Ce_0.7_Y_0.1_Yb_0.1_O_3-δ_ (Ni-BZCYYb) was tested under wet CO_2_ atmosphere. The hydrogen flux kept stable in wet 30 vol% CO_2_. As shown in Figure 6, no microstructure change happened on the membrane surface after the stability test. The authors attributed the excellent performance of the membrane to the Zr, Yb co-doping, and the modified membrane preparation method [45]. The effect of wet CO_2_ on cermet dual-phase membrane is complicated because of the existence of reverse water gas shift (RWGS) reaction. On one hand, more H_2_O generated by RWGS reaction can accelerate the protonic conductivity of the protonic conductors; on the other hand, the reaction between the ceramic and CO_2_ also can be promoted by H_2_O, which will lead to performance degradation of the membrane. Besides, due to forming CO in the feed side, metal particles expansion and membrane structure reconstruction caused by metal corrosion can also damage the membrane performance.

## 4. Cercer Dual-Phase Membrane

Owing to the combination of a metal phase and a ceramic protonic conductor, the mixed protonic-electronic conductivity of cermet dual-phase membranes is significantly improved compared with ceramic single-phase membranes. However, cermet membranes still exhibit some unresolved issues, such as the oxidation of the metal phase under high temperatures and the thermal expansion coefficient mismatch between metal and ceramic. In order to overcome the drawbacks of cermet membranes, researchers developed another kind of dual-phase membrane (cercer membranes), in which a ceramic with high electronic conductivity replaces the metal as the electronic conducting phase. Because both the electronic and protonic conducting phases of the membrane are ceramic, the chemical and thermal property compatibility of the membrane can be effectively improved. Unemoto first proposed the concept of cercer dual-phase membrane and investigated the electronic conductivity and hydrogen permeation properties of a model membrane, SrZrO_3_-SrFeO_3_ [59]. At present, cercer dual-phase materials are mainly based on barium cerate and lanthanum tungstate protonic conductors.

### 4.1. Barium Cerate-Based Cercer Membranes

As one of the advanced hydrogen-permeation materials, doped BaCeO_3_ is the most commonly used protonic conducting phase in cercer membranes and has the highest protonic conductivity among the oxide protonic conductors. In addition, it also presents a nonnegligible electronic conductivity especially at high temperatures, and its electronic conductivity is the highest among the perovskite protonic conductors [46,60]. So far, the main electronic conducting phases used for barium cerate cercer membranes include fluorite oxides Y, Gd- or Sm-doped CeO_2_, Nb-doped SrTiO_3_, and SrFeO_3_.

Rosensteel et al. designed a type of cercer hydrogen separation membrane using Y-doped BaCeO_3_ (BCY) and CeO_2_ (CYO) as the protonic and electronic conducting phase respectively. Fully dense dual-phase membranes were prepared via a single synthesis and sintering step. As a good electron conductor, CYO largely enhanced the membrane electronic conductivity [61]. Ivanova et al. also reported a cercer membrane BaCe_0.8_Eu_0.2_O_3-δ_-Ce_0.8_Y_0.2_O_2-δ_ (BCEO-CYO), which exhibited a remarkable high hydrogen permeation flux up to 0.61 mL·min^−1^·cm^−2^ at 700 °C. It was observed that Eu had a strong tendency to migrate from the BCEO phase to CYO phase, leading to a significant Eu-depleted BCEO [62]. The electronic conductivity of three kinds of electronic conducting phases at 800 °C follows the trend of Sr_0.95_Ti_0.9_Nb_0.1_O_3-δ_ (200 S·cm^−1^) > Sm_0.8_Y_0.2_O_2-δ_ (0.39 S·cm^−1^) > Ce_0.8_Y_0.2_O_2-δ_ (0.35 S·cm^−1^) [63,64,65]. Several representative cercer dual-phase membranes and their hydrogen permeation fluxes are listed in Table 2.

**Figure 7:**
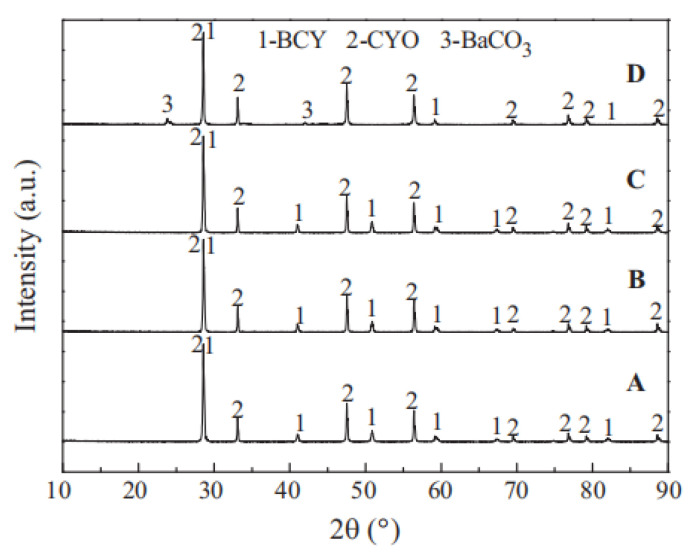
XRD patterns of BCY-CYO after the stability test in atmosphere containing 4% H_2_ (**B**), H_2_O (**C**), 100% CO_2_ (**D**); original membrane (**A**) [66].

The protonic conductivity of doped BaCeO_3_ is excellent. Theoretically, its chemical stability should be poor under CO_2_ atmosphere because there is a reaction between BaCeO_3_ and CO_2_ at high temperatures. However, as shown in the XRD images of BCY-CYO after the stability tests (Figure 7), compared with BaCeO_3_ single-phase membranes, the carbonate compounds generated reduced greatly under CO_2_ atmosphere, and as a result, the chemical stability of the membrane was significantly improved. Liu et al. further speculated the improvement of chemical stability attributed to the formation of CeO_2;_ CeO_2_ inhibited the reaction between CO_2_ and BaCeO_3_. As shown in Equation (4), the presence of CeO_2_ makes the reaction proceed to the direction of forming BaCeO_3_, thus protecting the perovskite structure [66].

(4) $${\mathrm{BaCe}}_{0.8}Y_{0.2}O_{3}+{\mathrm{CO}}_{2}⇌{\mathrm{BaCO}}_{3}+{0.8\mathrm{CeO}}_{2}+{0.1Y}_{2}O_{3}$$

### 4.2. Lanthanum Tungstate Cercer Membranes

**Figure 8:**
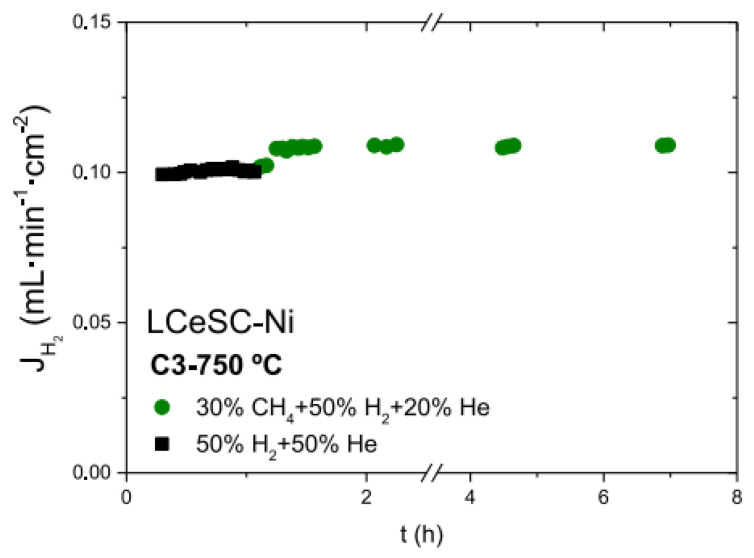
Hydrogen fluxes as a function of time for 60LWO-40LSC modified by La_0.75_Ce_0.1_Sr_0.15_CrO_3-δ_ (LCeSC)-Ni feeding with different gases [77].

Lanthanide tungstate oxides (LnWO, Ln = La, Nd, In, Sm) are important potential candidates used for hydrogen-separation membranes. These compounds present single defective fluorite structures while the ratio of Ln/W is in the range of 5.3 to 5.7. Besides the good protonic conductivity, lanthanide tungstate oxides exhibit better chemical stability against H_2_O, CO_2_, and H_2_S than perovskite materials. Lanthanum tungstate (LWO) is one of the main lanthanide tungstate oxides; this material has significant hydrogen permeability at temperatures above 800 °C, but with insufficient permeability at temperatures less than 700 °C because of the low electronic conductivity. Escolástico et al. prepared a La_5.5_WO_11.25-δ_-La_0.87_Sr_0.13_CrO_3-δ_ (LWO-LSC) dual-phase membrane using LSC, in which LSC, used as the electronic conductivity phase, had both high p-type electronic conductivity and remarkable stability in reducing atmosphere. The dense membrane was obtained by sintering at 1400 °C and the H_2_ flux at 700 °C reached 0.15 mL·min^−1^·cm^−2^, exhibiting a higher permeability than most of the BaCeO_3_-based cercer membranes [75]. The combination of LWO and LSC solved the abovementioned problem of LWO single-phase membranes well. Besides these, the stability and proton transport of composite membrane LWO-LSC was investigated under CH_4_ atmosphere. Hydrogen flux remained unchanged for 5 h when 30% CH_4_ was contained in the feed gas (Figure 8). The membrane stability under high steam content methane was also evaluated. After annealing at 700 °C and 3 bars for 24 h in 50% CH_4_-H_2_O atmosphere, the membranes kept totally dense and no secondary phase was found [77]. 

Polfus et al. also reported a densely sintered lanthanum tungstate dual-phase membrane: La_27_W_3.5_Mo_1.5_O_55.5-δ_-La_0.87_Sr_0.13_CrO_3-δ_. Either sweeping with wet or dry gas, the composite material showed enhanced hydrogen permeability compared with the single-phase material at all temperatures; especially the enhancement of the hydrogen flux was more significant below the temperature of 750 °C [78].

In conclusion, lanthanide tungstate membranes have attractive hydrogen permeability no less than perovskite membranes, especially under intermediate temperatures; moreover, their stability under harsh conditions such as high steam concentration or methane containing atmosphere makes them promising for applications in membrane reactors.

### 4.3. Elimination of the Inter-Phase Reaction

**Figure 9:**
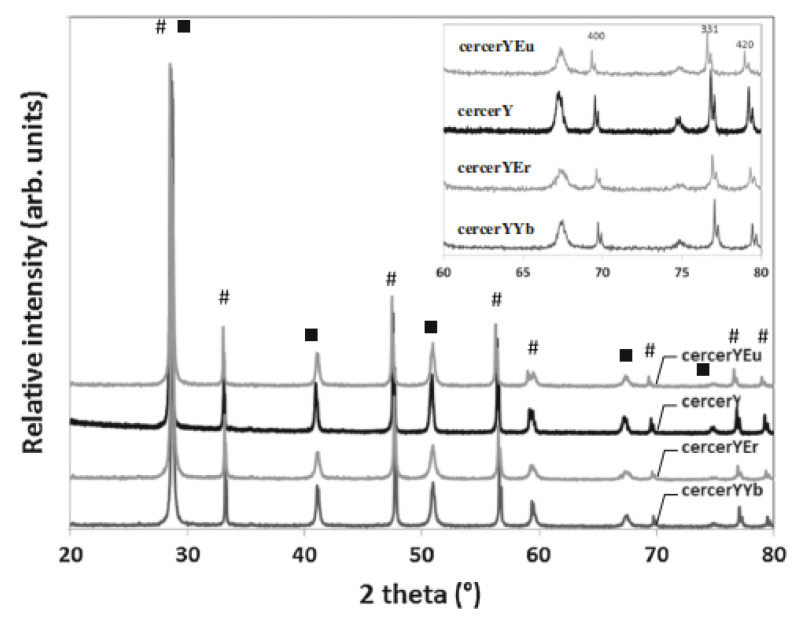
XRD of sintered cercer Y, cercer YYb, cercer YEu, and cercer YEr membranes. ■, perovskite phase; #, fluorite phase [65].

To obtain a dense membrane, a high-temperature sintering process is inevitable for the preparation of composite membranes. However, high temperatures tend to cause inter-phase reactions inside the membrane, generating undesirable phases which may damage the performances of the membrane. Adding sintering aid is an efficient method to decrease the sintering temperature, and then decrease the inter-phase reaction. Rebollo prepared BaCe_0.65_Zr_0.20_Y_0.15_O_3-δ_-Ce_0.83_Gd_0.15_O_2-δ_ dual-phase membrane via a single synthesis and sintering step using ZnO as the sintering aid. Dense membranes without any cracks were obtained by sintering treatments at 1450 °C. The addition of the sintering aid decreases the sintering temperature and avoids the detrimental of high temperature to the membrane mechanical and electrical properties [68]. Meng et al. fabricated a dual-phase membrane using ZnO and SrCe_0.95_Y_0.05_O_3-δ_. ZnO was used as both sintering aid and electronic conductor in the membrane. The membrane was sintered at 1200 °C and there was no new phase formed during the sintering progress. The membrane possessed the highest conductivity when the content of ZnO reached 20 wt% [73]. Wang et al. synthesized Zn doped BaZr_0.9_Y_0.1_O_3-δ_-BaCe_0.86_Y_0.1_Zn_0.04_O_3-δ_ dual-phase membranes by gel polymerization method; attributed to the doping of Zn, the membranes were well-sintered at 1350 °C and no extra phase was generated. In contrast, the non-doped membranes still kept their porous structure even when sintered at 1500 °C [81]. Updating sintering technologies also is one of the important methods to decrease the sintering temperature and eliminate the inter-phase reaction. Fish et al. synthesized BaCe_0.2_Zr_0.7_Y_0.1_O_3-δ_-Sr_0.95_Ti_0.9_Nb_0.1_O_3-δ_ dual-phase membranes using a popular material-processing method, technique-spark plasma sintering (SPS), instead of conventional solid-state reactive sintering (CS). The prominent advantage of SPS is that it can lower the membrane maximum temperatures by hundreds of degrees and reduce the sintering times to the order of minutes. The results showed that SPS was more effective than CS, and the sintering temperature of the membrane was lowered from 1500 to 1300 °C. Because the heating rate was fast and the sintering time was short, SPS samples showed high density and few inter-phase reactions. In contrast, CS samples had more reactions between phases and still remained their porous structure despite a higher sintering temperature being used [63]. In addition to the abovementioned method, Ricote et al. proposed to decrease the inter-phase reaction through reducing the cations number of the membrane materials. They prepared three different cercer membranes based on M, Y-codoped barium cerate BaCe_0.8_Y_0.1_M_0.1_O_3-δ_ and M, Y-codoped ceria Ce_0.8_Y_0.1_M_0.1_O_2-δ_, M = Yb, Er, and Eu. XRD, SEM results showed that dense samples were obtained without any cation segregation (Figure 9), and M, Y cations distributed uniformly across the membrane [65]. The materials with similar composition used can effective reduce the inter-phase reaction of cercer membranes and ensure the stability of the membrane.

### 4.4. Phase Composition Ratio

For cercer dual-phase membrane, both high protonic and electronic conductivity are necessary to obtain high hydrogen permeability, yet the phase ratio of the membranes have a great influence on the protonic and electronic conductivity. Therefore, to ensure high and comparable protonic and electronic conducting ability, the phase ratio of the membranes should be carefully investigated. 

Escolástico et al. prepared different composite membranes using La_5.5_WO_11.25-δ_ (LWO) and La_0.87_Sr_0.13_CrO_3-δ_ (LSC); the phase ratio was set as 2/8, 3/7, 4/6, and 7/3 respectively. The results showed that the 20%LWO-LSC composite had the highest total conductivity while the 50%LWO-LSC composite exhibited the highest hydrogen permeability [75]. Polfus et al. prepared La_27_W_3.5_Mo_1.5_O_55.5-δ_-La_0.87_Sr_0.13_CrO_3-δ_ (LWMO-LSC) cercer composite membranes with different phase ratios. The results showed that the best permeability was obtained by the membrane of 70LWMO-30LSC in which complete electronically conducting phase percolation might not be formed [78]. Similar to LWMO-LSC membrane, BCY-CYO membrane with phase ratio of 3/7 possessed the highest total conductivity and highest hydrogen permeability [66]. It indicates that the electronic or protonic conducting phase in composite membrane may not be necessary to form a continuous network, which has ever been considered needed for high hydrogen permeability. Adding too much electronic conducting phase not only has no benefit to the hydrogen permeability but also can choke off the conductivity of the protonic conducting phase. The hydrogen permeability enhancement should be attributed to the effective combination of the protonic and electronic phases, which brings about high ambipolar conductivity.

## 5. Combination Mode of the Protonic and Electronic Phases 

The distribution of protonic and electronic phases is also very important to the performance of dual-phase membranes, in addition to the phase ratio. Only by the organic combination of two phases can the membrane be densely sintered easily and the diffusion paths of the protons and electrons formed effectively. The combination mode of the phases mainly can be generalized powder blending, automatic phase separation, and independent distribution.

### 5.1. Powder Blending

Generally, most of the dual-phase membranes were prepared by powder-blending method; each phase powder is synthesized separately and then the two powders are mixed homogeneously by mechanical mixing. Ball milling is a mechanical technique to grind powders into fine particles. Two kinds of raw powders with certain amounts are put into a planetary ball mill, and milled for a certain time at a fixed milling speed. The particle size becomes refined and the microstructure of the particles becomes homogeneous after the ball milling. Ball milling possesses many advantages such as ease of operation, cost-effectiveness, and reproducibility. However, it also has some disadvantages including poor mixing effect, long milling time, and possibility of contamination. In order to improve the ball-milling effect, Kim et al. developed a high-energy milling technique to fabricate dual-phase membranes. Compared with ball milling, high-energy ball milling uses high-speed rotation balls with strong motion energy to grind powders and can reduce the particle size more effectively. Therefore, the mixed NiO-BCY powder by high-energy milling was finer and more homogeneous and the membrane microstructure was more uniform. The membrane hydrogen flux reached 0.76 mL·min^−1^·cm^−2^ at 800 °C, which was three times that of the membrane prepared via the conventional ball milling [48]. 

### 5.2. Automatic Phase Separation 

**Figure 10:**
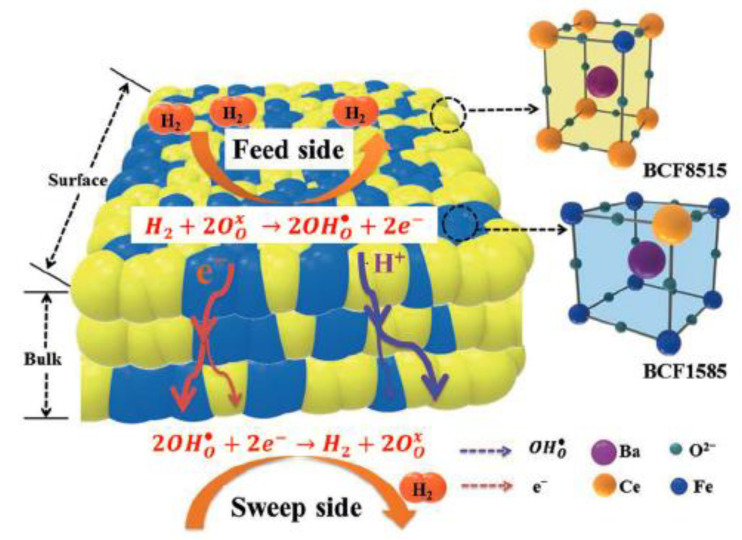
BaCe_0.5_Fe_0.5_O_3-δ_-based dual-phase membrane prepared by automatic phase separation technique [71].

To avoid cation diffusion and inter-phase reaction, a concept of automatic phase separation is proposed to prepare dual-phase membrane. Wang et al. first prepared BaCe_0.85_Fe_0.15_O_3-δ_-BaCe_0.15_Fe_0.85_O_3-δ_ dual-phase membrane using an automatic phase separation method [71]. The preparation of the membrane did not start with the mixing of the precursor powders, and even the precursor powders were not synthesized in advance. As a matter of fact, the membranes were formed by the auto-decomposition of the compound BaCe_0.5_Fe_0.5_O_3-δ_ at high temperatures. Because of the same chemical elements, the two phases of the membrane were thermodynamically stable and no extra phase was found between them in the repeated heating cycles. The formed protonic and electronic conducting phases distributed continuously and uniformly across the entire membrane (Figure 10). Because of the good match of the transport ability for protons and electrons, the membranes exhibited a very high ambipolar conductivity. The permeation flux reached 0.76 mL·min^−1^·cm^−2^ at 950 °C, which was one of the highest fluxes obtained by dual-phase membranes. Jia et al. also prepared a composite membrane by automatic phase separation technique; the well-distributed and good percolation degree of the auto-formed phases ensure high hydrogen permeability [72].

### 5.3. Independent Distribution 

**Figure 11:**
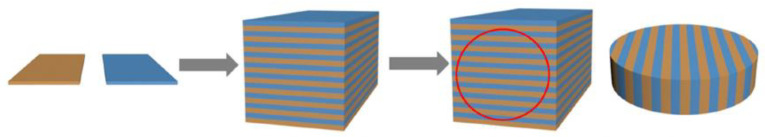
The schematic diagram preparing independent distributed dual-phase membranes [74].

**Figure 12:**
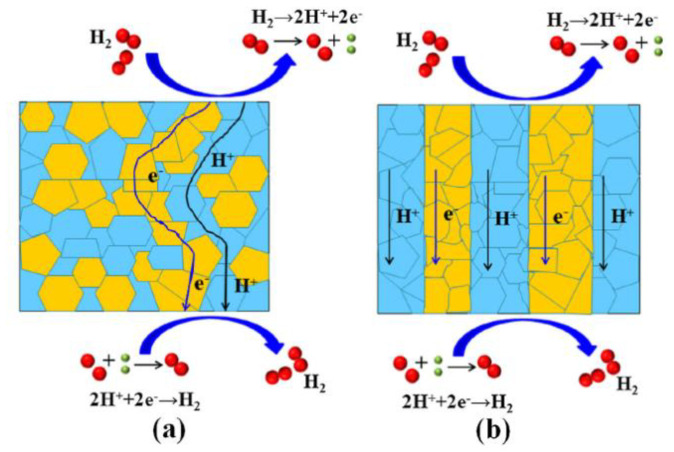
The schematic diagram of the phase distribution and the transport routs of protons and electrons, (**a**) dual-phase membrane prepared by simply powder blending, (**b**) laminated dual-phase membrane with short circuit pathways [74].

Diffusion resistance inside the membrane is one of the important factors affecting the permeability of MPEC membranes. Reducing the diffusion resistance can definitely accelerate the transport of the protons and electrons so as to improve the hydrogen flux. Whether the protonic and electronic conducting phases are combined by powder blending or automatic phase separation, the two phases are distributed randomly inside the membrane and the transport routes of the protons and electrons are flexuous, much longer than the membrane thickness. To decrease the diffusion resistance inside the membrane, Meng et al. designed an independently distributed dual-phase membrane via tape-casting technology. As shown in Figure 11, both protonic conductor SrCe_0.9_Y_0.1_O_3_ (SCY) and electronic conductor Ce_0.8_Sm_0.2_O_2_ (SDC) were cast into thin film layers and the two layers were alternately stacked into a block; the block was cut along the cross-section into thin slices, and then the dual-phase membranes were obtained via uniaxial pressing and sintering the thin slices. Short-circuit pathways were formed along with the longitudinal direction of the membrane; the diffusion paths of protons and electrons were largely decreased and the interface between the two phases was remarkably reduced (Figure 12). As a result, the membrane showed excellent hydrogen permeability, significantly higher than the SCY-SDC membrane prepared by conventional single synthesis [74].

## 6. Permeability Enhancement through Membrane Configuration Optimization

**Figure 13:**
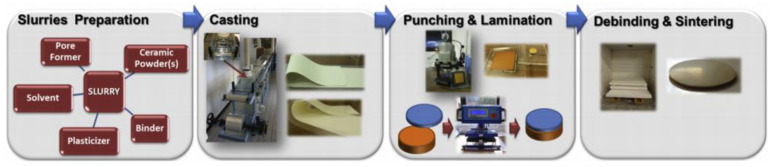
The preparation procedure of the asymmetric BCZY-GDC dual-phase membrane [69].

Besides composition optimization, configuration optimization is also an alternative method to enhance the performance of hydrogen permeation membranes. Asymmetric configuration is widely used for single-phase membranes to pursue higher hydrogen flux. With the support of the porous layer, the membrane dense layer can be made as thin as possible, decreasing the bulk diffusion resistance significantly. Similarly, composite membranes with asymmetric configuration are also developed to gain higher hydrogen permeability (listed in Table 1 and Table 2). In order to reduce the inter-phase reaction and thermal expansion mismatch, the dense and support layers of the asymmetric dual-phase membrane usually use similar materials. Additionally, the porous structure of the support layer is usually made through the combustion of the pore-forming agent such as activated charcoal or cornstarch. The fabrication methods of the asymmetric dual-phase membrane include dry pressing, tape casting, dip-coating, and coextrusion. Using tape a casting technique, Mercadelli et al. prepared an asymmetric dual-phase membrane using dense BaCe_0.65_Zr_0.2_Y_0.15_O_3-δ_-Ce_0.8_Gd_0.2_O_2-δ_ (BCZY-GDC) as the separation layer and BCZY-GDC with a porous structure as the support layer [69]. Rice starch was used as the pore former in the support layer, and the sintering performance of the membrane was improved by ZnO. The preparation conditions of the support layer were optimized from the amount of rice starch, organic content, and solid loading. Green discs of the dense and support layer were stacked to a BCZY-GDC bilayer, and then the asymmetrical BCZY-GDC membranes were formed through a press-and-sintering process. The dense layer thickness was as low as 20 µm that made the membrane possess higher hydrogen permeability than most of the all-ceramic membranes. The schematic representation of the tape casting process preparing asymmetric membrane is presented in Figure 13.

Meng et al. prepared Ni-BCTb asymmetric membranes using dry-pressing method [46]. Some soluble starch was added into Ni-BCTb powder and then the mixed powder was pre-pressed in a die under a certain pressure. The other part of Ni-BCTb powder for the dense layer was put onto the support layer uniformly and then co-pressed to obtain the membrane precursor. After sintering at high temperatures, the asymmetric cermet membranes with 90 µm-thick dense layers were prepared. At 850 °C, when 50% H_2_-N_2_ mixture was used in the feed side, the H_2_ flux reached 0.914 mL·min^−1^·cm^−2^. In contrast, the H_2_ fluxes of 650 µm-thick Ni-BCTb symmetric membrane and 300 µm-thick BCTb single-phase membrane were only 0.53 and 0.202 mL·min^−1^·cm^−2^ respectively [82]. Asymmetric composite membranes have a significant enhancement of hydrogen permeability compared with symmetric or single-phase membranes.

Owing to the distinct asymmetric structure, the application of hollow-fiber membranes in hydrogen separation has become more and more attractive over the past years. Hollow-fiber membranes have more advantages than disk-shaped or tubular membranes, for example, they have a larger specific surface area which is beneficial to gain higher separation efficiency, and they are easy to seal at high temperatures. Furthermore, because the dense and support layers are made by co-extrusion technique, cracks or the peeling-off phenomenon can be eliminated to a great extent. Considering the excellent performance of BCY-CYO, Cheng et al. fabricated a BCY-CYO hollow-fiber asymmetric dual-phase membrane via coextrusion technique. BCY-CYO was mixed with agglomerant, solvent, and stirred to form the homogeneous dense layer spinning suspension; the BCY-NiO support layer suspension was prepared by the same method. The hollow fiber precursors were created by the coextrusion of BCY-CYO and BCY-NiO suspensions using triple-orifice spinneret. The porous structure of the support layer was formed by the reduction of NiO in H_2_-containing atmosphere. The dense layer was attached to the support layer and the thickness reduced to 17 μm. At 900 °C, a hydrogen flux of 0.566 mL·min^−1^·cm^−2^ was reached when feeding with 50%H_2_-He. The membrane showed an excellent permeability at medium-low temperatures, for example, the hydrogen flux at 500 °C reached 0.065 mL·min^−1^·cm^−2^ [67]. 

## 7. Challenges and Future Directions 

### 7.1. Challenges

The main challenge of the dual-phase hydrogen permeation membranes is that the hydrogen permeability should be further improved. Although great efforts have been made to enhance the performance and the permeability is truly improved largely compared with the single-phase membrane, the hydrogen permeation flux of most of the dual-phase membranes is less than 1 mL·min^−1^·cm^−2^, which is still insufficient for practical applications. It is concluded from the literature that the protonic conductivity of most of the dual-phase membranes is lower than their electronic conductivity, the protonic transport process limiting the hydrogen permeation of the membrane. Therefore, it is challenging to improve the protonic conductivity of the protonic conducting phase of the membrane or to develop novel materials with high mixed protonic–electronic conductivity.

Second, a new preparation method should be developed. Now, the preparation methods of dual-phase membrane used are either with complex procedures or with high cost. Solid-state reaction is a high-cost method with multistep processes. Sol-gel route needs a complicated and time-consuming step for complete complexation. In order to gain homogeneous and dense membranes, milling and high-temperature sintering processes are usually inevitable, both of which are high energy consuming. Therefore, to adapt to large-scale applications, advanced preparation methods with simple procedures and low cost should be developed.

### 7.2. Future Directions

Materials with high protonic conductivity or high electronic conductivity should be further developed. Aliovalent doping is an efficient method to increase the oxygen vacancies of the protonic conducting phase; adjusting the kind and quantity of the doping ion both can improve the membrane hydrogen permeability. Besides the commonly used metal cation, metalloid anion or cation can also be used as the dopant to the protonic conductors. Wang et al. synthesized a phosphorus cation-doped hydrogen permeation membrane, La_5.5_W_0.6_Mo_0.35_P_0.05_O_11.25−δ_, and the protonic conductivity as well as the hydrogen permeation performance of the membrane were improved remarkably due to the P doping [83]. At present, considering the avoiding of the inter-phase reactions, the electronic conducting phases used for cercer membranes mainly focus on several ceramics such as doped ceria and lanthanum chromate. More materials with high electronic conductivity should be developed, and the inter-phase reactions can be eliminated by optimizing the membrane-preparation conditions, such as decreasing the sintering temperatures through adding sintering aids. In addition, inter-phase reactions are not necessarily harmful to the performance of the membrane, e.g., composite membrane BaCe_0.85_Fe_0.15_O_3-δ_-BaCe_0.15_Fe_0.85_O_3-δ_ was prepared from the decomposition reaction of the membrane precursor [71]. 

Besides the development of material, hydrogen permeability of composite membranes can also be enhanced through engineering perspectives. The surface reaction kinetics can be promoted by modifying the membrane with a catalyst and the bulk diffusion process can be shortened using a thinner membrane, both of which benefit to improve the hydrogen permeability of the membrane. In addition, the adjustment of the operation conditions is also useful to enhance the membrane permeability. Increasing the operation temperature can accelerate the transport of protons and improve membrane surface kinetics. Sweeping with humid gas is proved to be an efficient approach to accelerate the permeation of hydrogen through the MPEC membranes. Therefore, future research of dual-phase membranes can focus on the optimization of engineering perspectives and the adjustment of the operation conditions. The combination of material developing, membrane configuration optimization, and operation condition adjustment can greatly improve the performance of dual-phase membranes.

The hydrogen permeation mechanisms need further understanding. Composite membranes have a more complicated ion transport process compared with single-phase membranes; therefore, the mechanism of proton and electron transport and the role of the two phases acting in the hydrogen-permeation process need to be further investigated. It was suggestion that the electronic conducting phase in dual-phase membrane should form separate electronically conducting phase percolation in order to gain efficient electronic conductivity. Therefore, the proportion of the electronic conducting phase in the membrane composite should reach at least 50%. However, some experiments showed the different results, for example, the 70LWMO-30LSC membrane had the highest hydrogen permeability among the membranes with different phase ratios. Therefore, the hydrogen permeation mechanisms of the dual-phase membranes need further understanding and the respective role of the protonic conducting phase and electronic conducting phase of the membranes should be further investigated so as to guide the selection and matching of the materials. Generally, the optimization of the phase ratio and the investigation of the operation conditions both need masses of time-consuming experiments. Theoretical modeling can assist to determine the optimal experimental conditions and thus simplify the experimental process. 

## Figures and Tables

**Table 1:** Cermet membranes with different hydrogen permeation performances.

Composition	Hydrogen Flux (mL·min^−1^·cm^−2^)	T (°C)	Thickness (mm)	Feed/Sweep Gas	Note	Ref.
50Pd-50YSZ	20	900	0.22	100%H_2_/N_2_	mechanical mixing	[33]
50Pd-50YSZ	5.5	900	0.218	80% H_2_-He/N_2_	ball-milling	[36]
50Pd-50YSZ	52	900	0.018	100%H_2_/N_2_	mechanical mixing; asymmetric	[37]
50Pd-50Gd_0.2_Ce_0.8_O_2-δ_	5.5	900	0.282	80% H_2_-He/N_2_	ball-milling	[38]
50Pd-50CaZr_0.9_Y_0.1_O_3-δ_	2.3	900	0.50	80% H_2_-He/N_2_	ball-milling	[34]
50Pd-50BaCe_0.4_Zr_0.4_Gd_0.1_Dy_0.1_O_3-δ_	2.77	700	0.40	100%H_2_/N_2_	ball-milling	[35]
40Ni-60BaZr_0.7_Pr_0.1_Y_0.2_O_3-δ_	0.0162	950	0.40	wet50%H_2_-N_2_/Ar	mechanical mixing	[39]
50Ni-50BaCe_0.85_Te_0.05_ Zr_0.1_O_3-__δ_	0.17	800	0.50	50%H_2_-He/Ar	mechanical mixing	[40]
40Ni-60BaZr_0.1_Ce_0.7_Y_0.2_O_3-δ_	0.805	900	0.266	100%H_2_/N_2_	ball-milling	[41]
40Ni-60BaZr_0.1_Ce_0.7_Y_0.2_O_3-δ_	0.056	900	1	100%H_2_/N_2_	ball-milling	[42]
40Ni-60BaZr_0.1_Ce_0.7_Y_0.2_O_3-δ_	0.087	900	0.5	wet4%H_2_-Ar/N_2_	ball-milling	[43]
40Ni-60BaZr_0.1_Ce_0.7_Y_0.1_Yb_0.1_O_3-δ_	0.0174	900	0.75	20%H_2_-He/N_2_	mechanical mixing	[44]
40Ni-60BaZr_0.1_Ce_0.7_Y_0.1_Yb_0.1_O_3-δ_	0.215	900	0.40	wet80%H_2_-He/N_2_	mechanical mixing	[45]
50Ni-50BaCe_0.95_Tb_0.05_O_3-δ_	0.53	850	0.65	50%H_2_-He/N_2_	ball-milling	[46]
50Ni-50BaCe_0.95_Tb_0.05_O_3-δ_	0.914	850	0.09	50%H_2_-He/N_2_	ball-milling; asymmetric	[46]
40Ni-60BaCe_0.9_Y_0.1_O_3-δ_	0.24	900	0.08	wet3.8%H_2_-He/N_2_	ball-milling	[47]
40Ni-60BaCe_0.9_Y_0.1_O_3-δ_	0.76	800	0.23	100%H_2_/N_2_	high-energy milling	[48]
50Ni-50BaCe_0.85_Fe_0.15_O_3-δ_	0.325	1000	0.50	50%H_2_-He/wet Ar	ball-milling	[49]
40Ni-60BaCe_0.7_In_0.2_Ta_0.1_O_3-δ_	0.15	900	1	20%H_2_-N_2_/Ar	mechanical mixing	[50]
40Ni-60BaCe_0.7_Y_0.2_In_0.1_O_3-δ_	0.013	850	0.86	20%H_2_-N_2_ /Ar	mechanical mixing	[51]
40Ni-60SrCe_0.8_Yb_0.2_O_3-δ_	0.105	900	0.25	wet20%H_2_-He/N_2_	ball-milling	[52]
40Ni-60La_0.5_Ce_0.5_O_2-δ_	0.088	900	0.048	20%H_2_-N_2_/Ar	mechanical mixing; asymmetric	[53]
40Ni-60La_0.5_Ce_0.5_O_2-δ_	0.021	900	0.6	wet20%H_2_-N_2_/Ar	ball-milling	[54]
40Ni-60La_0.4875_Ca_0.0125_Ce_0.5_O_2-δ_	0.025	900	0.6	wet20%H_2_-N_2_/Ar	ball-milling	[54]
40Ni-60La_1.95_Sm_0.05_Ce_2_O_7_	0.037	900	0.6	wet20%H_2_- Ar/N_2_	ball-milling	[55]
60Ni-40La_5.5_WO_11.25-δ_	0.18	1000	0.5	50%H_2_-He/Ar	ball-milling	[56]
Cu-BaZr_0.9_Y_0.1_O_3-δ_	0.00242	882	1.90	100%H_2_/Ar	molten-copper infiltration technique	[57]
60Ta-40YSZ	1.2	500	0.50	100%H_2_/Ar	mechanical mixing	[58]

**Table 2:** Cercer dual-phase membranes and the hydrogen-permeation performances.

Composition	H_2_ Flux (mL·min^−1^·cm^−2^)	T (°C)	Thickness (mm)	Feed/Sweep Gas	Note	Ref.
50BaCe_0.8_Y_0.2_O_3-δ_-50Ce_0.8_Y_0.2_O_2-δ_	0.0744	900	1.44	wet50%H_2_-He/wet Ar	symmetric	[61]
30BaCe_0.8_Y_0.2_O_3-δ_-70Ce_0.8_Y_0.2_O_2-δ_	0.228	850	1.15	40%H_2_-N_2_/He	symmetric	[66]
30BaCe_0.8_Y_0.2_O_3-δ_-70Ce_0.8_Y_0.2_O_2-δ_	0.566	900	0.017	50%H_2_-He/N_2_	asymmetric	[67]
50BaCe_0.65_Zr_0.2_Y_0.15_O_3-δ_-50Ce_0.85_Gd_0.15_O_2-δ_	2.4	1040	0.65	50%H_2_-He/wet Ar	symmetric; ZnO	[68]
50BaCe_0.65_Zr_0.2_Y_0.15_O_3-δ_-50Ce_0.8_Gd_0.2_O_2-δ_	0.47	750	0.77	50%H_2_-He/Ar	asymmetric; ZnO	[69]
50BaCe_0.8_Eu_0.2_O_3-δ_-50Ce_0.8_Y_0.2_O_2-δ_	0.61	700	0.5	50%H_2_-He/wet Ar	symmetric	[62]
50BaCe_0.2_Zr_0.7_Y_0.1_O_3-δ_-50Sr_0.95_Ti_0.9_Nb_0.1_O_3-δ_	0.035	800	1	50%H_2_-He/Ar	symmetric; Pd coated	[70]
50BaCe_0.85_Fe_0.15_O_3-δ_-50BaCe_0.15_Fe_0.85_O_3-δ_	0.76	950	1	50%H_2_-He/Ar	auto-decomposition	[71]
50SrCe_0.95_Fe_0.05_O_3-δ_-50SrFe_0.95_Ce_0.05_O_3-δ_	0.38	940	0.7	60%H_2_-N_2_/wet Ar	auto-decomposition	[72]
80SrZrO_3_-20SrFeO_3_	0.0048	900	1	50%H_2_-He/wet Ar	symmetric	[59]
90SrCe_0.95_Y_0.05_O_3-δ_-10ZnO	0.039	900	1	21%H_2_-He/N_2_	symmetric	[73]
SrCe_0.9_Y_0.1_O_3_-Ce_0.8_Sm_0.2_O_2_	0.163	900	1	20%H_2_-He/N_2_	laminated	[74]
50La_5.5_WO_11.25-__δ_-50La_0.87_Sr_0.13_CrO_3-__δ_	0.15	700	0.37	wet50%H_2_-N_2_/wet Ar	symmetric	[75]
50La_5.5_WO_11.25-__δ_-50La_0.8_Sr_0.2_FeO_3-__δ_	0.15	900	0.5	50%H_2_-N_2_/wet Ar	symmetric	[76]
60La_5.5_WO_11.25-__δ_-40La_0.87_Sr_0.13_CrO_3-__δ_	0.22	750	0.3	wet50%H_2_-N_2_/wet Ar	symmetric; Pt coated	[77]
70La_27_W_3.5_Mo_1.5_O_55.5-δ_-30La_0.87_Sr_0.13_CrO_3-δ_	0.0077	700	1.43	wet50%H_2_-N_2_/wet Ar	symmetric; Pt coated	[78]
70La_27_W_3.5_Mo_1.5_O_55.5-δ_-30La_0.87_Sr_0.13_CrO_3-δ_	0.014	900	0.05	50%H_2_-He/Ar	asymmetric	[79]
70La_0.995_Ca_0.005_Nb_4-δ_-30LaNb_3_O_9_	0.0012	900	2	wet H_2_/wet Ar	symmetric	[80]
65Nd_5.5_WO_11.25-__δ_-35Cu_0.5_Ni_0.5_O	0.033	900	0.15	10% ethanol-40% H_2_O-Ar/Ar	asymmetric; catalyst coated	[76]

## Data Availability

Not applicable.
